# C677T Gene Polymorphism of MTHFR Is a Risk Factor for Impaired Renal Function in Pregnant Women With Preeclampsia in the Chinese Han Population

**DOI:** 10.3389/fcvm.2022.902346

**Published:** 2022-05-30

**Authors:** Lin Yun, Meiqi Ge, Rui Xu, Fei Zheng, Xueqiang Zhao, Xinran Li

**Affiliations:** ^1^Shandong Provincial Qianfoshan Hospital, Shandong University, Jinan, China; ^2^Department of Medicine, Jinan Maternity and Child Care Hospital, Jinan, China; ^3^Department of Cardiology, The First Affiliated Hospital of Shandong First Medical University & Shandong Provincial Qianfoshan Hospital, Shandong Medicine and Health Key Laboratory of Cardiac Electrophysiology and Arrhythmia, Jinan, China

**Keywords:** pregnancy hypertension, preeclampsia, impaired renal function, *MTHFR*, risk factor

## Abstract

Impaired renal function in pregnant women with preeclampsia is particularly common, yet there is no consensus about implementation. This lack of consensus is due in part to uncertainty about risks for disease progression. Limited evidence suggests that C677T gene polymorphism of 5, 10-methylenetetrahydrofolate reductase (*MTHFR* C677T) may affect impaired renal function in pregnant women with preeclampsia in Chinese Han population. To investigate the association between *MTHFR* C677T and impaired renal function in pregnant women with preeclampsia, a total of 327 pregnant women diagnosed with gestational hypertension (GH) or preeclampsia-eclampsia (PE) from January 2016 to December 2021 were selected as the study subjects. The personal information, gestational information, clinical indicators, and the C677T gene polymorphism of *MTHFR* were tested. Compared with the GH group, the PE renal function impairment group had increased in blood pressure, homocysteine level, liver and kidney function indicators (creatinine, uric acid, urea nitrogen, cystatin C, alanine aminotransferase, aspartate aminotransferase, cholyglycine), and blood lipids (total cholesterol, triglycerides and low density lipoprotein) but had reductions in plasma protein (total protein, albumin, globulin, prealbumin), trace elements (calcium and zinc), prothrombin time and fibrinogen. The homocysteine level in the TT genotype was higher than that in the CC and CT genotypes. Binary logistic regression analysis showed that the *MTHFR* C677T gene polymorphism was associated with PE renal function impairment in the recessive model (OR: 1.620, 95% CI: 1.033–2.541, *P* < 0.05). These findings show that the C677T gene polymorphism of *MTHFR* is an independent risk factor for impaired renal function in pregnant Chinese Han women with PE.

## Introduction

Hypertensive disorders of pregnancy (HDP), including gestational hypertension (GH), preeclampsia-eclampsia (PE), pregnancy-associated chronic hypertension and chronic hypertension with superimposed preeclampsia (1), are idiopathic diseases of pregnancy, posing serious threats to the health of mothers and infants. The incidence of PE in developing countries such as China is higher than that in developed countries (2). Since pregnancy is a special physiological process, hypertension and other diseases with specific occurrences in pregnancy deserve special attention. Different from primary hypertension, the basic pathological changes of HDP include systemic arteriolospasm, which leads to poor blood flow in the organs throughout the body, insufficient blood supply in the microcirculation, and damage of tissues and organs due to ischemia and hypoxia. Among these factors, impaired renal function is particularly common. Impaired renal function is very important in the assessment and treatment of HDP because its occurrence mechanism in HDP is not exactly equal to the renal damage caused by chronic hypertension. Therefore, the pathogenesis of impaired renal function in HDP has gradually gained attention from the research field.

Genetic factors play an important role in the occurrence and development of HDP. For example, *CCR5* gene polymorphism is associated with PE in Brazilian women (3); endothelial nitric oxide synthase (*eNOS*) gene polymorphism is associated with PE in Egyptian women (4); and *CDH13* gene polymorphism is associated with PE in Han women (5). However, based on the current literature in China and abroad, there are no studies identifying the susceptibility genes for impaired renal function in HDP or PE, and there are few studies conducting comprehensive analyses of the pathogenesis of impaired renal function in HDP or PE based on genetic and environmental factors.

Combined plasma homocysteine elevation is a special feature of the hypertensive population in China. Due to genetic and environmental factors, the average plasma homocysteine level in Chinese adults with primary hypertension is 15 μmol/L, and approximately 75% of patients have elevated plasma homocysteine levels (6). Elevated plasma homocysteine is associated with the C677T gene polymorphism of 5,10-methylenetetrahydrofolate reductase (*MTHFR*) (7), which is the key enzyme in the metabolism of homocysteine. The frequency of the mutant T allele of the *MTHFR* gene polymorphism C677T is high, present in 41% of the Chinese population (8). Our previous study confirmed that plasma homocysteine was associated with early renal impairment in Chinese Han patients with primary hypertension (9). It is possible that the C677T gene polymorphism of *MTHFR* may also affect the occurrence of impaired renal function among Chinese Han pregnant women with HDP, which has not been investigated previously.

Due to the different pathogenic mechanisms of HDP and primary hypertension, this study focused on pregnant women with GH and PE and did not include pregnant women with chronic hypertension. Urinary protein is recognized as an important indicator of impaired renal function. Therefore, this study used urinary protein as a marker of impaired renal function in PE patients. We used the case-control method and detected plasma homocysteine and the C677T gene polymorphism in its key metabolic enzyme *MTHFR*, urinary albumin, and multiple clinical indicators possibly associated with HDP to analyze the risk factors of impaired renal function in pregnant Chinese Han women with PE. This study intended to explain the reasons for the high incidence of renal function impairment in pregnant Chinese Han women with PE from a new perspective and to provide a theoretical basis for the early prevention and treatment of renal function impairment in pregnant women with PE in clinical practice.

## Methods

### Enrolled Populations

A total of 327 pregnant women diagnosed with GH or PE in our hospital from January 2016 to December 2021 were selected as the study subjects. We recorded the patients' age, height, weight, history of smoking, gestational week, gestational week upon the occurrence of elevated blood pressure, family history of hypertension, HDP history, and other related personal information. Among them, the gestational week was defined as the gestational week at the end of pregnancy; gestational week upon the occurrence of elevated blood pressure was defined as the gestational week at the time of the first diagnosis of HDP; a family history of hypertension was defined as the presence of immediate family members with a history of hypertension; and a history of HDP was defined as the diagnose of HDP during prior pregnancies.

### Grouping Criteria

Inclusion criteria: GH was defined as the first occurrence of hypertension after 20 weeks of gestation, with a systolic blood pressure ≥140 mmHg and/or a diastolic blood pressure ≥90 mmHg, which returned to normal within 12 weeks postpartum, and with negative results in urinary protein tests. PE was defined as the first occurrence of systolic blood pressure ≥140 mmHg and/or diastolic blood pressure ≥90 mmHg after 20 weeks of gestation, with random urinary protein ≥ (+) or any organ or systemic involvement. We used random urinary protein, which is commonly used clinically and is easy to detect, as a basis for grouping based on impaired renal function. Since random urinary protein ≥(2+) is considered to indicate the presence of impaired renal function (1), only PE patients with random urinary protein ≥(2+) were enrolled in this study.

Exclusion criteria: (1) secondary hypertension; (2) pregnancy complicated with chronic hypertension, or chronic hypertension with superimposed preeclampsia; (3) renal parenchymal or vascular lesions; (4) severe heart failure or liver and kidney failure; (5) obstetric and gynecological acute and critical illness (amniotic fluid embolism, etc.); (6) tumor; (7) recent serious infections; and (8) multiple organ dysfunction syndrome.

Grouping criteria: (1) GH group (159 cases): systolic blood pressure ≥140 mmHg and/or diastolic blood pressure ≥90 mmHg and negative random urine protein results; (2) PE renal function impairment group (168 cases): systolic blood pressure ≥140 mmHg and/or diastolic blood pressure >90 mmHg and prenatal random urine protein ≥(2+).

### Laboratory Methods

Urine protein was measured using the Siemens BN ProSpec special protein analyzer. Plasma homocysteine was determined using the Oppland OP-162 micro-fluorescence detector. Liver and kidney function, blood glucose, and blood lipids were detected using the Hitachi MODULAR PP automated biochemical analyzer. Trace elements were measured using the Tiancheng TC-3010B trace element analyzer.

In total, 4 ml peripheral blood samples from subjects were collected and stored at 4°C. Genomic DNA was extracted from the samples using Blood DNA System (NOBELAB BIOTECHNOLOGIES CO, LTD, Beijing) and stored at −80°C for later use. Then, the *MTHFR* C677T (rs1801133) was genotyped with the direct DNA sequencing method. The primer sequences were as follows: 5′-CAA GCA ACG CTG TGC AAG TTC TGG-3′and 5′-TGT GCT GTG CTG TTG GAA GGT GCA-3′. PCR amplification was performed. DNA was denatured at 95°C for 5 min, amplified by 40 s cycles at 95°C for 30 s and cooled at 58°C for 30 s, 72°C for 1 min, and a final elongation at 72°C for 5 min. For SNP rs1801133, the PCR products were sequenced by DNA sequencing. The inner primers were used for the cycle-sequencing reaction, and genotyping was analyzed using an ABI3730XL DNA sequencer.

### Statistical Methods

SPSS 17.0 software was used for statistical analysis. Measurement data are expressed as the means ± standard deviations ($\bar{x}$ ± *s*). Counting data are expressed as proportions (%). Comparisons between means of two groups were conducted using the t test. Comparisons between means of three groups were conducted using one-way analysis of variance. Pair-wise comparisons between two groups were conducted using the Bonferroni test. Count data were conducted using the chi-square (χ^2^) test. Gene distribution was tested using the Hardy–Weinberg equilibrium test. The relationship between disease and gene polymorphism was analyzed by binary logistic regression. *P* < 0.05 indicated that the difference was statistically significant.

## Results

### Comparison of Basic Information Between the Two Groups

There were 159 cases in the GH group, with a mean age of (31.46 ± 5.76) years. There were 168 patients with impaired renal function in the PE group, with a mean age of (31.78 ± 5.08) years. There were no significant differences in age, body mass index (BMI), history of smoking, family history of hypertension, history of HDP, infant gender, twin ratio, number of pregnancies, and number of births between the two groups (*P* > 0.05).

Compared with the GH group, the PE renal function impairment group had an earlier gestational week at the end of pregnancy (37.18 ± 2.59 weeks, 33.92 ± 3.64 weeks, *P* < 0.01) and an earlier gestational week upon the occurrence of elevated blood pressure (34.84 ± 4.32 weeks, 32.54 ± 4.42 weeks, *P* < 0.01) and had an increased proportion of cesarean sections for pregnancy termination (60.4, 92.9%, *P* < 0.01). In addition, the PE renal function impairment group had a lower neonatal body weight (3,023.27 ± 641.28 g, 2,340.06 ± 816.29 g, *P* < 0.01) and Apgar score (9.32 ± 0.57, 8.78 ± 1.14, *P* < 0.01) than those in the GH group. The details are shown in Table 1.

**Table 1:** Comparison of basic information between the two groups.

**Item**	**GH group** **(*n* = 159)**	**PE group** **(*n* = 168)**	***P*** **-Value**
Age (year)	31.46 ± 5.76	31.78 ± 5.08	0.595
**Body mass index (kg/m** ^2^ **)**	31.08 ± 5.14	31.39 ± 4.07	0.552
<24	7.5%	2.4%	0.080
24–27.9	18.2%	16.7%	
≥28	74.2%	81.0%	
History of smoking (%)	1.9%	1.8%	0.946
Gestational week (week)	37.18 ± 2.59	33.92 ± 3.64^a^	0.000
GWEBP (week)	34.84 ± 4.32	32.54 ± 4.42^a^	0.000
Family history of hypertension (%)	3.8%	4.8%	0.787
History of HDP (%)	3.8%	8.9%	0.071
Cesarean sections (%)	60.4%	92.9%^a^	0.000
Infant gender (male%)	42.8%	50.6%	0.183
Neonatal body weight (g)	3,023.27 ± 641.28	2,340.06 ± 816.29^a^	0.000
Apgar score (score)	9.32 ± 0.57	8.78 ± 1.14^a^	0.000
Twin ratio (%)	8.8%	3.6%	0.064
**Number of pregnancies (%)**			
1	37.1%	25.0%	0.125
2	28.3%	28.6%	
3	16.4%	20.8%	
>3	18.2%	25.6%	
**Number of births (%)**			
0	52.8%	45.2%	0.141
>1	47.2%	54.8%	

*GH group, gestational hypertension group; PE group, preeclampsia-eclampsia renal function impairment Group; GWEBP, gestational week upon the occurrence of elevated blood pressure; HDP, hypertensive disorders of pregnancy*.

a *P < 0.01*.

### Comparison of Clinical Biochemical Indicators Between the Two Groups

Clinical biochemical indexes between the two groups were compared by t test. Compared with the GH group, the PE renal function impairment group showed increases in the systolic blood pressure (142.53 ± 16.18, 157.29 ± 18.84 mmHg, *P* < 0.01), diastolic blood pressure (96.26 ± 12.17, 100.57 ± 11.42 mmHg, *P* < 0.01), homocysteine (12.20 ± 6.84, 16.58 ± 9.88 μmol/L, *P* < 0.01), creatinine (46.56 ± 13.62, 54.18 ± 15.49 μmol/L, *P* < 0.01), uric acid (307.31 ± 92.35, 391.47 ± 89.37 μmol/L, *P* < 0.01), urea nitrogen (3.62 ± 1.10, 4.82 ± 1.79 mmol/L, *P* < 0.01), cystatin C (1.08 ± 0.33, 1.41 ± 0.37 mg/L, *P* < 0.01), alanine aminotransferase (10.62 ± 5.83, 15.26 ± 14.30 U/L, *P* < 0.05), aspartate aminotransferase (18.90 ± 8.07, 23.32 ± 11.56 U/L, *P* < 0.05), cholyglycine (1.64 ± 1.40, 2.66 ± 3.16 mg/L, *P* < 0.01), total cholesterol (6.01 ± 1.45, 6.81 ± 1.63 mmol/L, *P* < 0.01), triglycerides (3.12 ± 1.40, 4.05 ± 1.98 mmol/L, *P* < 0.01), low density lipoprotein (3.07 ± 0.94, 3.96 ± 1.21 mmol/L, *P* < 0.01), serum magnesium (0.83 ± 0.23, 0.91 ± 0.32 mmol/L, *P* < 0.05), and serum phosphorus (1.19 ± 0.24, 1.32 ± 0.18 mmol/L, *P* < 0.01), but reductions in white blood cell (11.78 ± 3.98, 10.74 ± 2.78 10^9^/L, *P* < 0.01), serum total protein (59.77 ± 5.97, 52.93 ± 5.97 g/L, *P* < 0.01), serum albumin (33.27 ± 3.99, 28.78 ± 3.49 g/L, *P* < 0.01), serum globulin (26.29 ± 3.54 g/L, 24.15 ± 3.45 g/L, *P* < 0.01), serum prealbumin (181.05 ± 44.98, 145.60 ± 38.78 mg/L, *P* < 0.01), calcium (2.20 ± 0.17, 2.05 ± 0.26 mmol/L, *P* < 0.01), zinc (9.06 ± 2.60, 7.29 ± 1.95 μmol/L, *P* < 0.01), total bilirubin (7.41 ± 5.39, 5.62 ± 3.09 μmol/L, *P* < 0.01), direct bilirubin (2.82 ± 1.30, 2.35 ± 0.99 μmol/L, *P* < 0.01), indirect bilirubin (4.59 ± 4.52, 3.24 ± 2.36 μmol/L, *P* < 0.01), superoxide dismutase (154.57 ± 25.62, 133.70 ± 25.82 U/L, *P* < 0.01), alkaline phosphatase (166.14 ± 211.66, 126.92 ± 54.19 U/L, *P* < 0.05), prothrombin time (11.82 ± 0.69, 11.50 ± 0.76 s, *P* < 0.01), and fibrinogen (4.78 ± 2.16, 3.98 ± 1.07 mg/L, *P* < 0.01). The remaining indicators showed no significant differences (*P* > 0.05). The details are shown in Table 2.

**Table 2:** Comparison of clinical biochemistry indicators between the two groups.

**Item**	**GH group** **(*n* = 159)**	**PE group** **(*n* = 168)**	***P*** **-Value**
Systolic blood pressure (mm Hg)	142.53 ± 16.18	157.29 ± 18.84^a^	0.000
Diastolic blood pressure (mm Hg)	96.26 ± 12.17	100.57 ± 11.42^a^	0.000
Homocysteine (μmol/L)	12.20 ± 6.84	16.58 ± 9.88^a^	0.000
White blood cell (10^9^/L)	11.78 ± 3.98	10.74 ± 2.78^a^	0.006
Hemoglobin (g/L)	109.45 ± 20.11	111.23 ± 16.36	0.382
Platelet (10^9^/L)	215.66 ± 64.60	204.46 ± 63.96	0.117
Ferritin (ng/ml)	53.03 ± 27.28	48.32 ± 29.75	0.370
Serum creatinin (μmol/L)	46.56 ± 13.62	54.18 ± 15.49^a^	0.000
Uric acid (μmol/L)	307.31 ± 92.35	391.47 ± 89.37^a^	0.000
Blood urea nitrogen (mmol/L)	3.62 ± 1.10	4.82 ± 1.79^a^	0.000
Cystatin C (mg/L)	1.08 ± 0.33	1.41 ± 0.37^a^	0.000
Fasting plasma glucose (mmol/L)	5.22 ± 1.26	5.12 ± 1.43	0.533
Total protein (g/L)	59.77 ± 5.97	52.93 ± 5.97^a^	0.000
Albumin (g/L)	33.27 ± 3.99	28.78 ± 3.49^a^	0.000
Globulin (g/L)	26.29 ± 3.54	24.15 ± 3.45^a^	0.000
Prealbumin (mg/L)	181.05 ± 44.98	145.60 ± 38.78^a^	0.000
Alanine aminotransferase (U/L)	10.62 ± 5.83	15.26 ± 14.30^a^	0.001
Aspartate aminotransferase (U/L)	18.90 ± 8.07	23.32 ± 11.56^a^	0.000
Total bilirubin (μmol/L)	7.41 ± 5.39	5.62 ± 3.09^a^	0.002
Direct bilirubin (μmol/L)	2.82 ± 1.30	2.35 ± 0.99^a^	0.001
Indirect bilirubin (μmol/L)	4.59 ± 4.52	3.24 ± 2.36^a^	0.005
Glutamyltranspetidase (U/L)	14.39 ± 30.31	15.59 ± 21.73	0.723
Superoxide dismutase (U/L)	154.57 ± 25.62	133.70 ± 25.82^a^	0.000
Alkaline phosphatase (U/L)	166.14 ± 211.66	126.92 ± 54.19^b^	0.028
Creatine kinase MB (U/L)	8.72 ± 16.54	6.93 ± 9.19	0.349
Total bile acid (μmol/L)	3.35 ± 2.08	3.89 ± 4.05	0.196
Cholyglycine (mg/L)	1.64 ± 1.40	2.66 ± 3.16^a^	0.007
Serum calcium (mmol/L)	2.20 ± 0.17	2.05 ± 0.26^a^	0.000
Serum iron (μmol/L)	15.14 ± 9.86	14.31 ± 8.35	0.471
Serum zinc (μmol/L)	9.06 ± 2.60	7.29 ± 1.95^a^	0.000
Serum phosphorus (mmol/L)	1.19 ± 0.24	1.32 ± 0.18^a^	0.000
Serum magnesium (mmol/L)	0.83 ± 0.23	0.91 ± 0.32^b^	0.019
Total cholesterol (mmol/L)	6.01 ± 1.45	6.81 ± 1.63^a^	0.005
Triglycerides (mmol/L)	3.12 ± 1.40	4.05 ± 1.98^a^	0.005
Low-density lipoprotein (mmol/L)	3.07 ± 0.94	3.96 ± 1.21^a^	0.000
High-density lipoprotein (mmol/L)	1.62 ± 0.46	1.71 ± 0.45	0.263
Prothrombin time (s)	11.82 ± 0.69	11.50 ± 0.76^a^	0.001
Activated partial thromboplastin time (s)	25.76 ± 6.67	26.67 ± 4.02	0.201
Fibrinogen (g/L)	4.78 ± 2.16	3.98 ± 1.07^a^	0.000
d-Dimer (mg/L)	2.95 ± 4.56	4.56 ± 9.81	0.150

*GH group, gestational hypertension group; PE group, preeclampsia-eclampsia renal function impairment group*.

a *P < 0.01*.

b *P < 0.05*.

### MTHFR C677T Genotyping

**Figure 1:**
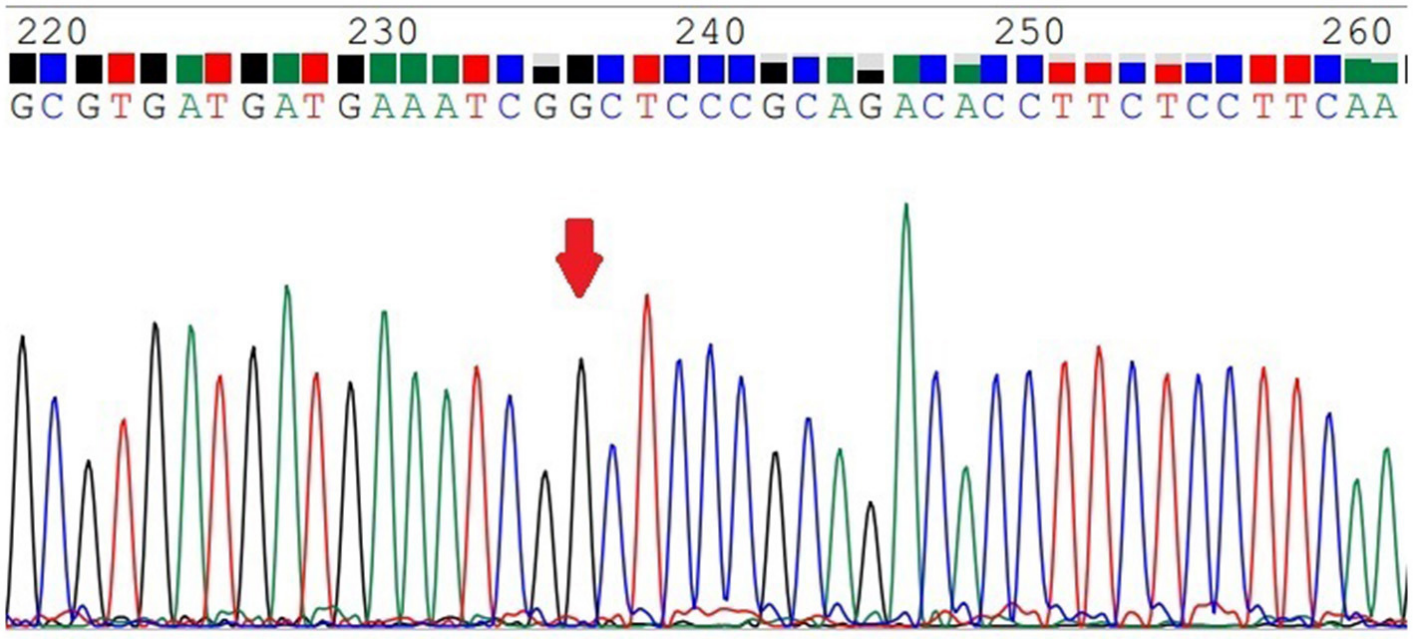
The *MTHFR*-C677T CC genotype.

**Figure 2:**
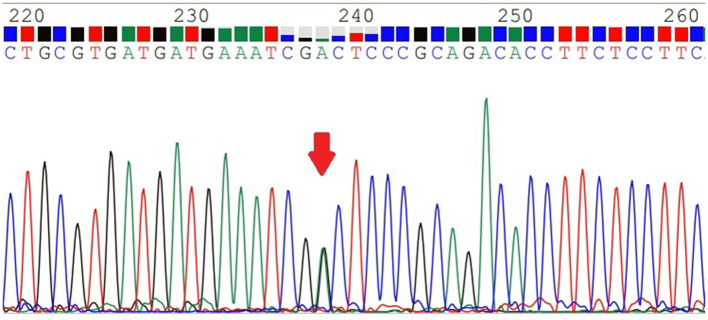
The *MTHFR*-C677T CT genotype.

**Figure 3:**
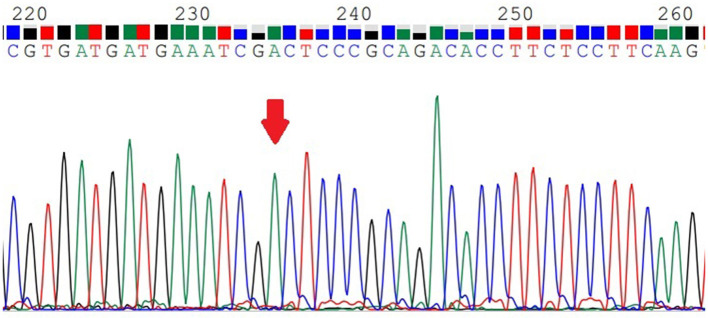
The *MTHFR*-C677T TT genotype.

*MTHFR* C677T was divided into three genotypes (Figures 1–3), including the CC genotype in 79 cases, the CT genotype in 122 cases, and the TT genotype in 126 cases. There were no significant differences between the three genotypes in terms of age and BMI (*P* > 0.05). One-way ANOVA analysis of plasma homocysteine in the three genotypes showed that the homocysteine level in the TT genotype was higher than that in the CC and CT genotypes (11.77 ± 5.22 μmol/L, 10.67 ± 4.01 μmol/L, 19.79 ± 11.06 μmol/L, *P* < 0.01). There was no significant difference between the CC and CT genotype groups (*P* > 0.05). The details are shown in Table 3.

**Table 3:** *MTHFR* C677T genotyping.

**Item**	**CC group** **(*n* = 79)**	**CT group** **(*n* = 122)**	**TT group (*n* = 126)**	***P*** **-Value**
Age (year)	31.89 ± 5.72	31.75 ± 4.71	31.34 ± 5.86	0.746
Body Mass Index (kg/m^2^)	31.79 ± 6.24	31.23 ± 3.44	30.90 ± 3.97	0.412
Homocysteine (μmol/L)	11.77 ± 5.22	10.67 ± 4.01	19.79 ± 11.06^ab^	0.000

*Compared with CC group*,

a *P < 0.01, Compared with CT group*,

b *P < 0.01*.

### Analysis of the Relationships Between *MTHFR* C677T Gene Polymorphism and Impaired Renal Function in PE

The distributions of genes in the GH group and the PE renal function impairment group were in accordance with Hardy-Weinberg equilibrium (*P* > 0.05, Table 4).

**Table 4:** Hardy-weinberg equilibrium testing.

**Group (*n*)**	**Allele**		***P*** **-Value**
	**C (%)**	**T (%)**	
GH group (*n* = 159)	147 (46.2%)	171 (53.8%)	0.086
PE group (*n* = 168)	133 (39.6%)	203 (60.4%)	

*GH group, gestational hypertension group; PE group, preeclampsia-eclampsia renal function impairment group*.

Binary logistic regression analysis showed that the *MTHFR* C677T gene polymorphism was associated with PE renal function impairment in the recessive model [odds ratio (OR): 1.620, 95% confidence interval (CI): 1.033–2.541, *P* < 0.05]. In the dominant and additive models, *MTHFR* C677T gene polymorphism had no correlation with PE renal function impairment (*P* > 0.05). The details are shown in Table 5.

**Table 5:** Analysis of the relationships between *MTHFR* C677T gene polymorphism and impaired renal function in PE.

***MTHFR*** **genotype**	**GH group (*n* = 159)**	**PE group (*n* = 168)**	**Recessive**		**Dominant**		**Additive**	
			**OR (95% CI)**	***P*** **-Value**	**OR (95% CI)**	***P*** **-Value**	**OR (95% CI)**	***P*** **-Value**
CC	40 (25.2%)	39 (23.2%)	1.620 (1.033–2.541)	0.036^a^	1.112 (0.670–1.845)	0.682	1.246 (0.942–1.649)	0.124
CT	67 (42.1%)	55 (32.7%)						
TT	52 (32.7%)	74 (44.1%)						

*GH group, gestational hypertension group; PE group, preeclampsia-eclampsia renal function impairment group; MTHFR, methylenetetrahydrofolate reductase; CI, confidenceinterval; OR, odd ratio*.

a *P < 0.05*.

## Discussion

Hypertensive disorders of pregnancy is an idiopathic disease that occurs in mid-stage or late-stage pregnancy, seriously damaging maternal and child health. The Chinese Han population has a relatively high incidence of hypertensive renal damage and additional, more prominent issues with impaired renal function in pregnant women with HDP. Pregnant women with PE are often in critical condition upon admission and require emergency cesarean sections; in such cases, it is not suitable to conduct prenatal collection for 24-h urine protein determination. Many postpartum PE patients recover quickly, and postpartum random urine protein testing or 24-h urine protein determination cannot truly reflect the patient's prenatal condition. Therefore, we used prenatal random urinary protein as a reference standard to determine the presence of impaired renal function in pregnant women with PE. This grouping mode shifted the research focus from the previous grouping mode of “mild preeclampsia, severe preeclampsia and eclampsia” to the grouping mode of “whether or not renal function is impaired,” with clearer aims and better research focus.

The age, BMI, history of smoking, family history of hypertension, history of HDP, sex of infants, proportion of twins, number of pregnancies, and number of births matched between the two groups that we selected had no significant differences between the groups (*P* > 0.05), ensuring the reliability of this study. Compared with the GH group, the PE renal function impairment group had an earlier gestational week at the end of pregnancy and an earlier gestational week upon the occurrence of elevated blood pressure and had an increased proportion of cesarean sections ending pregnancy. In addition, neonatal body weight and Apgar scores in the PE renal function impairment group were lower than those in the GH group, indicating that the occurrence of PE renal function impairment increases risks in pregnancy. To ensure the safety of mothers and infants, the cesarean section rate was increased, as was the premature birth rate, but the preterm infants' body weights and Apgar scores were lower. Therefore, investigating the pathogenesis of PE renal function impairment, identifying the risk factors, and conducting early prevention and treatment are conducive to improving maternal and child health.

Differing from the pathogenesis of primary hypertension, the current recognized pathogenic mechanisms of HDP include immune theory, placental ischemia theory, and theory of heredity. We explored the pathogenesis of renal damage in PE from the perspective of genetics combined with environmental factors to more comprehensively and objectively evaluate the early prediction value of gene polymorphism, which can provide the theoretical basis for future early clinical diagnosis and treatment. As mentioned above, the investigation of the relationship between the C677T gene polymorphism of *MTHFR*, a key enzyme of plasma homocysteine metabolism, and PE renal function impairment is based on the high mutation rate of the *MTHFR* T allele and the high hyperhomocysteinemia incidence in the Chinese population. Our previous study also confirmed that the *MTHFR* C677T gene polymorphism was associated with impaired renal function in a hypertensive Chinese Han population (9). Does the correlation of this genetic polymorphism still exist in the special group of pregnant Han Chinese women? We performed *MTHFR* genotyping of the populations enrolled in this study and found that homocysteine expression in the TT genotype group was higher than in the CC and CT genotype groups (*P* < 0.01), with no significant difference between the CC genotype and CT genotype groups (*P* > 0.05). We also conducted a binary logistic regression analysis of the relationships between genetic polymorphisms and impaired renal function in PE and found that in the recessive model, the C677T gene polymorphism of *MTHFR* was associated with impaired renal function in PE.

The above results indicate that the mutation of the T allele may be an independent risk factor for impaired renal function in Chinese pregnant women with PE. The C677T mutation is the most common missense mutation in *MTHFR*; it can occur stably in populations around the globe and has a worldwide distribution of high frequency. In normal populations, the frequency of TT homozygotes is approximately 4% in Egyptian (10) and approximately 23% in Italians in Europe (11), while the T allele frequency is at a higher level of 41% in the Chinese population (8). According to Hou (12), the T allele frequency of healthy pregnant women is 27%. But our study showed that the frequency of the T allele in the GH group was 53.8%, and the frequency of the T allele in the PE renal function impairment group was 60.4%, both of which were higher than those reported above and similar to the findings of Ding et al. (13). As mentioned above, our previous study found that *MTHFR* C677T gene polymorphism is an important cause of renal damage in hypertensive Han Chinese patients.This feature still exists in Chinese Han pregnant women. This also proves that genetics plays an important role in this disease. The activity of normal *MTHFR* decreases by 60% after 5 min at 46°C, whereas the activity of the *MTHFR* mutant encoded by the C677T mutation decreases by 80%−90%. This change in thermolability leads to decreased *MTHFR* activity in the human body, which in turn results in an increase in plasma homocysteine concentration. A plasma homocysteine level above 10 μmol/L is known as hyperhomocysteinemia (14). Association with hyperhomocysteinemia is a special feature of Chinese hypertension populations. The patients included in this study had an average plasma homocysteine level above 10 μmol/L and a hyperhomocysteinemia rate of 75.2%, with rates of 73.0% in the GH group and 77.4% in the PE renal function impairment group. Hyperhomocysteinemia can damage glomerular capillary endothelial cells through a variety of mechanisms (15), affecting endothelial function, exacerbating urinary protein, and leading to impaired renal function, similar to the results of Li et al. (16). Therefore, we infer that the mutation rate of the T allele of *MTHFR* C677T is higher in Chinese Han PE patients with impaired renal function, affecting the metabolism of homocysteine and leading to an increase in the proportion of patients with hyperhomocysteinemia, in turn producing more urinary protein.

In addition to homocysteine expression and *MTHFR* C677T gene polymorphism, there are many factors affecting PE renal function impairment. This study also found that compared with the GH group, the PE renal function impairment group had increases in blood pressure (systolic blood pressure, diastolic blood pressure), liver and kidney function indicators (creatinine, uric acid, urea nitrogen, cystatin C, alanine aminotransferase, aspartate aminotransferase, cholyglycine), and blood lipids (total cholesterol, triglycerides and low-density lipoprotein) but had reductions in plasma protein (total protein, albumin, globulin, prealbumin), trace elements (calcium and zinc), prothrombin time and fibrinogen. These results are similar to the observations of Seremak-Mrozikiewicz et al. (17). Systemic arteriolospasm in PE patients leads to an increase in blood pressure, causing hyperperfusion, hyperfiltration, and high transmembrane pressure in the glomerulus, along with impaired glomerular endothelial cells and increased urinary protein secretion (18). Urine protein loss from the kidneys, coupled with insufficient liver synthesis of proteins, leads to impaired renal function in pregnant women with PE, often accompanied by hypoproteinemia and edema. Pregnant women with PE in severe conditions often experience ischemia and hypoxia in organs throughout the body on top of impaired liver and kidney functions. In our clinical practice, we found that the brain and myocardial cells were also damaged. Regular testing of liver and kidney function in patients with PE can lead to a dynamic understanding of the disease progress in patients. Trace elements such as calcium are involved in the regulation of a variety of physiological functions in the body. Hypocalcemia can lead to increased intracellular calcium concentrations and activation of myosin and myofibrillar proteins in vascular smooth muscle, thus causing arteriolar spasm, which is involved in the occurrence of HDP (19). Because of the demands of fetal growth and fat reserves, blood lipid levels are often elevated during pregnancy. However, a significant increase in blood lipid levels can inhibit anti-oxidation *in vivo* and can cause vasospastic contraction, thus affecting the development of HDP (20).

Many other diseases are also associated with C677T gene polymorphism of *MTHFR*. Such as cardiovascular diseases, diabetes, venous thrombosis (21) and breast cancer (22). Hyperhomocysteinemia is an emerging risk factor for various cardiovascular diseases. Folate and vitamin B_12_ are key elements of the one-carbon metabolism pathway in which *MTHFR* matters, supplementation of which may help reduce homocysteine levels (23). Different doses of folate have different efficacy of lowering homocysteine in hypertensive Chinese adults. One study showed that, in patients with hyperhomocysteinemia, 0.4 mg/day folate can significantly reduce the homocysteine level in CC genotype but at least 0.8 mg/day folate can reduced the homocysteine level in TT genotype (24). Therefore, if pregnant women with a high homocysteine level, the *MTHFR* C677T genotypes should be tested and different doses of folate can be selected according to the different genotype. However, if homocysteine level is normal, screening for the *MTHFR* may be not necessary.

Although we comprehensively analyzed the relationships between the *MTHFR* gene polymorphism C677T, homocysteine, and various biochemical indicators of pregnancy and PE renal function impairment, we did not investigate whether the reduction in plasma homocysteine (such as with folic acid supplementation) can help to reduce urinary protein levels. We plan to investigate the pathways by which plasma homocysteine affects the impaired renal function from the perspective of molecular biology to provide a theoretical basis for the clinical prevention and treatment of renal function impairment in PE patients.

In summary, since the C677T polymorphism of the *MTHFR* gene, a key enzyme in plasma homocysteine metabolism, is an independent risk factor for impaired renal function in pregnant Chinese Han women with PE, we should conduct early detection of the *MTHFR* gene polymorphism C677T in this population and should control plasma homocysteine levels within the normal range. In addition, regular monitoring of blood pressure, liver and kidney functions, blood lipids, platelets, trace elements, and other indicators can lead to a dynamic understanding of PE renal impairment so that intervention can be conducted as early as possible to ensure the safety of mothers and infants.

## Data Availability Statement

The raw data supporting the conclusions of this article will be made available by the authors, without undue reservation.

## Ethics Statement

The studies involving human participants were reviewed and approved by the Ethics Committee of Shandong First Medical University. The patients/participants provided their written informed consent to participate in this study.

## Author Contributions

LY and MG acquired, analyzed and interpreted data, and wrote the manuscript. FZ and XZ reviewed and edited the manuscript. XL analyzed data. RX designed the study, acquired and analyzed, and interpreted the data. All authors read and approved the final manuscript.

## Funding

This study was funded by (1) National Science Foundation for Incubation Fund of Shandong Provincial Qianfoshan Hospital (Grant No. QYPY2020NSFC1011). This fund provides fees for article publication, etc. (2) Shandong Provincial Key Research and Development Programme Foundation, China (Grant No. 2018GSF118009). This fund provides genetic testing fees. (3) Technology Programme Foundation of Jinan, China (Grant No. 201805060). This fund provides patient testing and management costs.

## Conflict of Interest

The authors declare that the research was conducted in the absence of any commercial or financial relationships that could be construed as a potential conflict of interest.

## Publisher's Note

All claims expressed in this article are solely those of the authors and do not necessarily represent those of their affiliated organizations, or those of the publisher, the editors and the reviewers. Any product that may be evaluated in this article, or claim that may be made by its manufacturer, is not guaranteed or endorsed by the publisher.
